# Wild vegetable mixes sold in the markets of Dalmatia (southern Croatia)

**DOI:** 10.1186/1746-4269-9-2

**Published:** 2013-01-03

**Authors:** Łukasz Łuczaj, Marijana Zovko Končić, Tihomir Miličević, Katija Dolina, Marija Pandža

**Affiliations:** 1 Department of Botany and Biotechnology of Economic Plants, University of Rzeszów, Institute of Applied Biotechnology and Basic Sciences, Werynia 502, 36-100 Kolbuszowa, Poland; 2 Department of Pharmacognosy, University of Zagreb, Faculty of Pharmacy and Biochemistry, Marulicev trg 20, 10000 Zagreb, Croatia; 3 Department of Plant Pathology, University of Zagreb, Faculty of Agriculture, Svetošimunska 25, 10000 Zagreb, Croatia; 4 University of Dubrovnik, Institute for Marine and Coastal Research, Kneza Damjana Jude 12, 20000 Dubrovnik, Croatia; 5 Marija Pandža, Primary school "Murterski škoji", Put škole 8, Murter, 22243, Croatia

**Keywords:** Wild food plants, Herbophilia, Market survey, Ethnobotany, Ethnobiology, Wild edible plants

## Abstract

**Background:**

Dalmatia is an interesting place to study the use of wild greens as it lies at the intersection of influence of Slavs, who do not usually use many species of wild greens, and Mediterranean culinary culture, where the use of multiple wild greens is common. The aim of the study was to document the mixtures of wild green vegetables which are sold in all the vegetable markets of Dalmatia.

**Methods:**

All vendors (68) in all 11 major markets of the Dalmatian coast were interviewed. The piles of wild vegetables they sold were searched and herbarium specimens taken from them.

**Results:**

The mean number of species in the mix was 5.7. The most commonly sold wild plants are: *Sonchus oleraceus* L., *Allium ampeloprasum* L., *Foeniculum vulgare* Mill., *Urospermum picroides* F.W.Schmidt, *Papaver rhoeas* L., *Daucus carota* L., *Taraxacum* sp., *Picris echioides* L., *Silene latifolia* Poir. and *Crepis* spp. Also the cultivated beet (*Beta vulgaris* L.) and a few cultivated Brassicaceae varieties are frequent components. Wild vegetables from the mix are usually boiled for 20–30 minutes and dressed with olive oil and salt. Altogether at least 37 wild taxa and 13 cultivated taxa were recorded.

Apart from the mixes, *Asparagus acutifolius* L. and *Tamus communis* L. shoots are sold in separate bunches (they are usually eaten with eggs), as well as some Asteraceae species, the latter are eaten raw or briefly boiled.

**Conclusions:**

The rich tradition of eating many wild greens may result both from strong Venetian and Greek influences and the necessity of using all food resources available in the barren, infertile land in the past. Although the number of wild-collected green vegetables is impressive we hypothesize that it may have decreased over the years, and that further in-depth local ethnobotanical studies are needed in Dalmatia to record the disappearing knowledge of edible plants.

## Background

The use of wild green vegetables (leaves, buds, stalks etc.) is very widespread around the Mediterranean [1,2]. Although this culinary tradition has decreased due to economic changes in nutrition and agriculture, the contemporary use (at least by older people) of many species of wild greens has been documented in Italy [3-11], Iberian Peninsula [12-17] (but not among the Basque people [18]), Greece [19-21], Turkey (e.g. [22]) and Palestine [23]. The phenomenon of the wide use of wild leafy vegetables in nutrition was named *herbophilia*[24]. In northern Europe a much smaller number of species of wild greens was used and they were associated mainly with famine. This attitude was named *herbophobia*. It is however unclear how old this division of attitudes towards wild greens is, as, for example in Poland, the use of wild greens has undergone substantial changes (i.e. decreased) since the 17^th^ century [25]. Generally however, peasants from Slavic countries used to resort to just a few of the commonest wild greens, ignoring other species. Exceptions to this are some regions inhabited by southern Slavs, i.e. the inhabitants of Herzegovina [26,27]; and the coast of southern Croatia - Dalmatia [28], who seem to have used an exceptionally high number of wild leafy vegetables in nutrition, as pointed out by Moszyński [29]. Unfortunately, apart from Redžić’s works [27,30,31] there are no other English-language publications documenting the use of wild food plants in western Balkans. Newer and newer works are published on the ethnobotany of this area, e.g. from Albania [32,33], Serbia [34,35], Kosovo [36,37] and Bosnia-Herzegovina [38]. Although there are also some studies about Croatia [39-41] and about the ancient Croatian diaspora in Italy [42], this country, one of the largest and most diverse in the region, seems to be the most neglected one.

There are a few publications on the use of wild food plants in Croatia. Ljubiša Grlić published a series of wild food guides (e.g. [28,43]). Although they belong to popular science literature he inserted many valuable observations on the use of particular species in Croatia, particularly in Dalmatia, often quoting concrete sub-regions or islands where a plant is used. Another source of information on the food ethnobotany of the Croatian coast is the work of Bakić and Popović [44], who organized a census of emergency foods used during World War II in coastal areas and islands, all the way from Istria to Dubrovnik. This work is based on an impressive number of 5000 questionnaires, and lists not only the food use of plants but also land and marine animals. Unfortunately only a list of the most commonly used organisms is included. Valuable information on the wild herbs eaten on the island of Korčula is also present in a conference paper by Sardelić [45].

In Dalmatia most wild greens are used in the form of a mix called *mišanca*, *mišancija*, *gruda*, *parapač*, *pazija*[30,45], commonly sold in vegetable markets. As the first part of documentation of the use of wild food plants we aimed at cataloguing wild food vegetables sold in these markets. Market research is a commonly applied approach in ethnobotany [46], also for studying the use of wild green vegetables (e.g. [47-49]). One of the pioneers of market research in ethnobiology was the Polish ethnographer Jerzy Szulczewski [50], who recorded the species of mushrooms sold in the markets of Poznań at the beginning of the 20^th^ century. Plants sold in markets are usually those with highest cultural value. People can easily be approached and interviewed. These methods also enabled the repetition of the study after some years in order to compare the changes in the plants’ availability in markets.

Our objective was to record the commonest wild food species of plants sold in vegetable markets in the form of mixes, their names, modes of preparations and origins. The results will be utilized in the future to compare them with local in-depth studies in chosen villages.

## Methods

As Dalmatia (outside the tourist season) is sparsely populated, vegetable markets are concentrated on the mainland coast, whereas in the islands only single wild vegetable vendors can be approached. Early spring (the second half of March), during the blooming of fruit trees and the appearance of asparagus shoots, was chosen as the time of study as this is, according to our preliminary information from the inhabitants of Dalmatia, the top season for selling wild vegetables in the markets.

**Figure 1:**
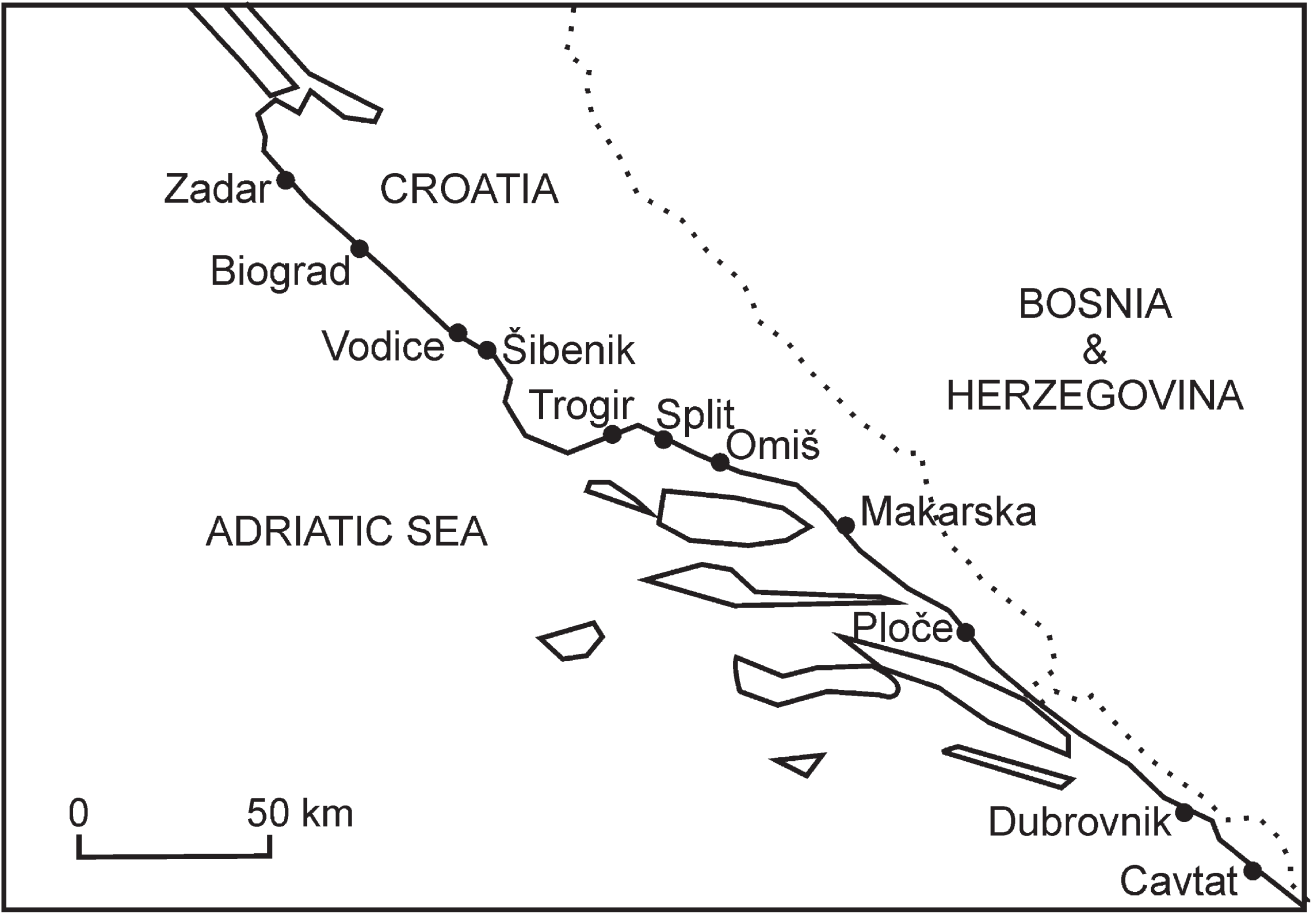
The map of the studied markets.

Possibly all the vegetable markets of the Dalmatian part of Adriatic coast were visited. These 11 markets (Figure 1) were visited once in the morning (between 8 and 11 am), apart from the market in Zadar which was visited twice. All the 68 sellers of wild vegetable mixes were interviewed. The research was carried out following the code of ethics of the American Anthropological Association ( http://www.aaanet.org/issues/policy-advocacy/upload/AAA-Ethics-Code-2009.pdf) and the International Society of Ethnobiology Code of Ethics (with 2008 additions, http://ethnobiology.net/code-of-ethics/). Oral prior informed consent was acquired. The sellers allowed us to search through the piles of plants they sold. The amount of information they supplied varied (some female informants refused to give us their age). The interviews were performed in the Croatian standard of the Serbo-Croatian language (often classified as a separate Croatian language). The piles of vegetables were photographed. Herbarium specimens were collected from the sold plants and occasionally sellers were interviewed with a bunch of flowering specimens collected in the same towns/villages, including plants sold in the markets and those which are not, to give a broader context of plant choice. During each interview we asked which species were collected from the wild and which were cultivated, paying particular attention to *Beta*, *Allium* and Brassicaceae species, which could have been of wild or of cultivated origin. Voucher specimens were collected and deposited in the herbarium of the Faculty of Biology of Warsaw University (WA). Precautions for the identification of plants in ethnobotanical studies outlined by Łuczaj [51] were taken into account. In the case of the specimens without flowers, we tried to identify them using vegetative parts sold and folk names. If the botanical names of plants given as the equivalents of the folk names from Šugar’s dictionary of Croatian plants names [52] and the other cited papers matched the morphological characteristics of the leaves we collected – such identifications were given but usually labelled with a question mark.

The average age of sellers is 63, median - 66.5. Most sellers are women (87%). Some of them are farmers and have been selling plants in the market since their childhood, others only after retirement. Nine men were also encountered, mostly farmers but also one retired restaurant chef. Except for one, who collected the plants himself, it was their wives who collected the mix. Most women sellers claimed that they collect the plants themselves, apart from three younger women who said that their older relatives do it for them. Nearly all the sellers were able to name every species found in the mix. The sellers come to the market well before 7 am and stay until 11 am – 1 pm.

The gathered information was also enriched by the personal experiences of the co-authors who have life-long personal experience with living/travelling around Dalmatia (T.M.– around Vrgorac, Imotski, Metković in Dalmatia and Ljubuški, Čitluk, Grude in SW Herzegovina; K.D. – Dubrovnik; M.P. – island of Murter and Šibenik; M.Z.K – Zadar and Grude, Široki Brijeg in SW Herzegovina). It is commonly accepted that research involving many-year participant experiences of the researcher (e.g. having spent childhood in the study area) is a very valuable contribution to an ethnobiological study [53,54].

The plant nomenclature follows Flora Europaea [55] with author abbreviations following The International Plant Name Index [56].

## Results

Wild vegetables are sold in all the vegetable markets of Dalmatia. Most sellers along with the mixes sell other plants, mainly home-grown vegetables, home-made olive oil and brandy. Most wild plants are sold in the form of a mix. Only *Asparagus acutifolius* L. (Croatian *šparoga*), *Tamus communis* L. (*kuka*, *kukoce*) and *Foeniculum vulgare* L. (*komorač*, *morač*) are sold in separate bunches (sometimes *A*. *acutifolius* and *T*. *communis* are mixed together). Occasionally a single Asteraceae species is sold separately, mainly *Taraxacum* sp. or *Crepis* sp. One seller of *Papaver rhoeas* L. shoots was encountered in Makarska. In Ploče no one sold a species-rich wild vegetable mix: one lady was selling a mix of *Sonchus* and *Papaver rhoeas* and three sellers were selling large bags of *Sonchus oleraceus* on its own.

Three main mix names were encountered during the study: *mišancija*/*mišanca*/ *mješancija* (means literally *the mix*, especially in western Dalmatia), *divlje zelje* (literally *wild herbs*, whole Dalmatia) and *pazija* (Turkish for beet, Dubrovnik).

**Figure 2:**
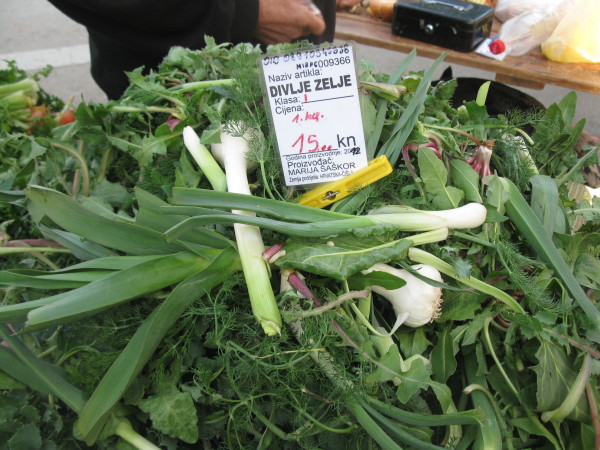
A wild vegetable mix in the market of Omiš.

**Figure 3:**
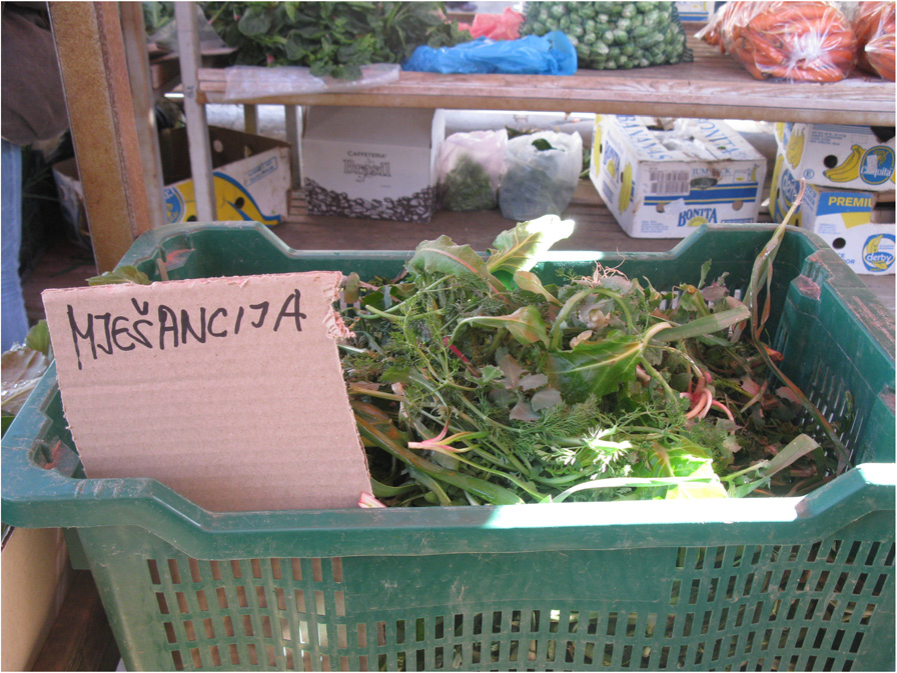
A wild vegetable mix in the market of Šibenik.

**Figure 4:**
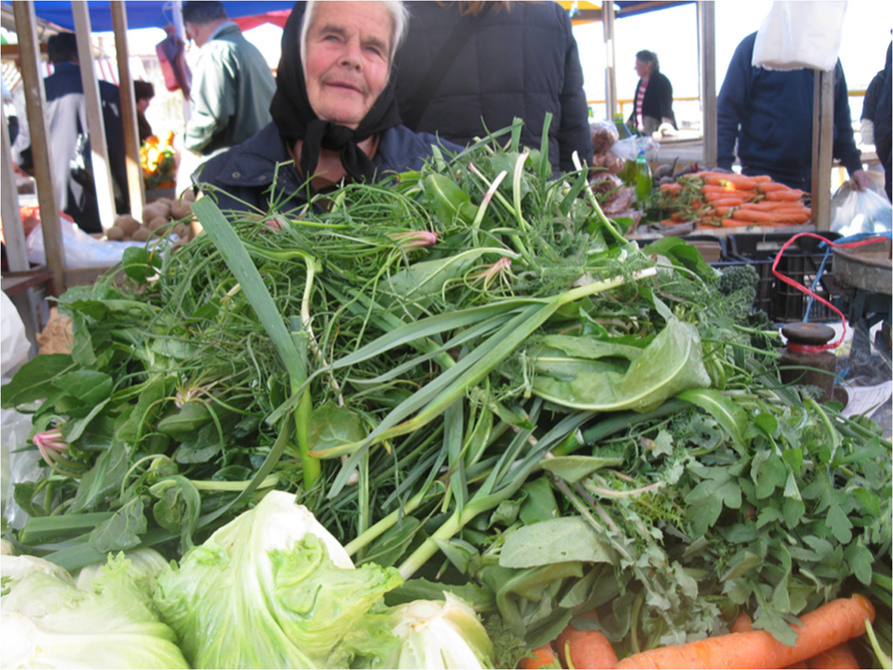
**One of the oldest sellers of wild herbs in Dalmatia(Šibenik), aged 80, who has been selling the mix for over 60 years.**

On average 5.7 species are sold by one seller (median 5, modal value 5). The average number of species in the mix slightly decreased eastwards: the Spearman rank correlation between the position of the market on the coast (Zadar – 1, Biograd - 2, … Dubrovnik – 9, Cavtat −10) and the number of species was *r* = −0.23, *P* = 0.064. The total list of plants consists of at least 50 taxa, of which 37 are collected from the wild. The sellers usually sell 1 to 4 kg of the mix per day, charging 10–20 kuna (1.6-3.2 USD) per kg (Figures 2, 3, 4). Most sellers come to the market regularly, at least once a week. They come from neighbouring villages.

Most of the bulk weight of the mix is composed of a few most commonly used species (Table 1). The composition is repeatable although often one to three of the commonest species are missing. The most often used species are sow thistles (*Sonchus* spp.), beet (*Beta vulgaris* L.), wild leek (*Allium ampeloprasum* L.), wild fennel (*Foeniculum vulgare* Mill.), prickly goldenfleece (*Urospermum picroides* F.W.Schmidt, this species only in the Dubrovnik area), bristly ox-tongue (*Picris echioides* L.), common poppy (*Papaver rhoeas* L.), wild carrot (*Daucus carota* L.), dandelion (*Taraxacum* sp.), white campion (*Silene latifolia* Poir.) and a group of Cichorioideae (Asteraceae) taxa called ž*utenica*/*radić* (mainly *Crepis* spp. and *Cichorium intybus* L.).

**Table 1:** List of taxa found in the wild vegetable mixes in Dalmatia in 2012

**Family**	**Botanical species**	**Folk names recorded**	**No. of stalls which sell it N** = **68**	**Markets**	**Voucher specimen no. (preceded by WA0000028)**
Alliaceae	*Allium ampeloprasum* L.	divlji luk, *also*: zečji luk, divlji poriluk, lukenj, lukovac, divlji poruk, lukovica	47	all but P, C	245
	*Allium ascalonicum* L.*	ljutika domaća, ljutika	2	M	-
	*Allium porrum* L.*	poriluk	3	O, T, Z	-
Amaranthaceae	*Beta vulgaris* subsp. *vulgaris* L. [cultivated]*	blitva	47**	all but P, C	248, 255, 283
	*Beta vulgaris* subsp. *maritima* (L.) Moq. (syn. *B*. *maritima* L.) [wild]	divlja blitva	1	T	-
	*Spinacia oleracea* L.*	špinat, špinjak, mličenjak	4	B, D, Z	-
Apiaceae	*Daucus carota* L. [wild, only shoots]	divlja mrkva, divlja mrkvica	13	B, D, M, SS, Z	281, 295
	*Eryngium campestre* L.	brmbeč, brmbečica, sikavac	2	B, Z	290
	*Foeniculum vulgare* Mill.	komorač, koromač, kumurač, morač, moroč, morača	43**	all but P, C	322
	*Levisticum officinale* L.	divlji selen	1	D	324
	*Pimpinella peregrina L*. (?)	vrati muž	1	D	282
	*Smyrnium olusatrum* L.	vrati muž, lesandra, divlja selen	1	D	284
	Apiaceae, not identified (not *Daucus*)	divlja mrkva	4	D, Z	287, 311
Asparagaceae	*Asparagus acutifolius* L.	šparoga	1**	D	250
Asteraceae	*Cichorium intybus* L.	žutenica domaća	3**	C, M	-
	*Lactuca serriola* L.	divlja salata	5	O, V, SS, Z	268, 307
	*Picris echioides* L. (mainly) and *Urospermum picroides* F.W.Schmidt	*P*. *echioides* (western Dalmatia): prelipača, lipovica, lipovic, lipovac, krasavica; both species (eastern Dalmatia): hrastej, rastej, pakolič, pakoleč, parotina	27	B, C, D, Z	278, 292, 312
	*Reichardia picroides* (L.) Roth. (?)	slačka	1	M	259
	*Scorzonera laciniata* L.	kozja brada	9	O, SS, V	269, 296
	*Scorzonera villosa* Scop. (?)	kozja brada, turutva	1	SS	-
	several Cichorioidae species, mainly *Crepis* spp. but also *Taraxacum* sp., *Leontodon* spp. and related genera	žutenica, žutanica, žutinica, žućenica, radič, radić	14**	D, O, T, S, SS	252, 256, 262, 266, 272,279, 285, 291, 297, 304, 308, 314, 320
	*Sonchus* spp. (mainly *S*. *oleraceus* L. but probably all the local taxa from the genus are eaten)	kostriš, kostrič, čevčeg, sušak, mišnjak, mličnjak, svinjak, četveg	54	all	246, 271, 305, 313
	*Taraxacum* sp.	maslačak	10**	O, T, V, SS, Z	258, 265, 277, 303
	*Tragopogon* sp.	kozja brada	1	V	-
	Asteraceae – unidentified, not *Tragopogon* and not *Scorzonera*	kozja brada	5	O, S, SS	302
Boraginaceae	*Borago officinalis* L.	borač	1	Z	-
Brassicaceae	*Brassica oleracaea* L. young leaves*	cimule, cimulice, kupus, kapusac	7	M, O, V, SS, Z	253, 257, 273
	*Brassica oleracea* L. Italica Group*	brokula, brokolica	2	SS, V	-
	*Brassica oleracea* L. ssp. *bullata* DC.*	kelj	1	V	-
	*Brassica rapa* L. [whole young plants with small roots]*	rodakva (r. domača)	3	SS	264, 309
	Brassicaceae (?), unidentified;	divlja repa	1	Z	-
	*Nasturtium officinale* L. (?) [this folk name is usually applied to *Diplotaxis* sp. in Dubrovnik]	divlja rikula	1	D	280
Caryophyllaceae	*Silene latifolia* Poir.	ušac, ušak, zečje uši, loboda	9	B, S, Z	247, 254, 298, 321
Dioscoreaceae	*Tamus communis* L.	bljušt, kuka	1**	O	299
Fabaceae	*Vicia faba* L.*	bob	1	C	-
Geraniaceae	*Erodium cicutarium* (L.) Ľ Hér.	vranja noga, iglica	3	SS	263
Lamiaceae	*Lamium amplexicaule* L.	*no folk name given by seller*	1	B	-
	*Melissa officinalis* L.*	matičnjak	1	B	-
Papaveraceae	*Papaver rhoeas* L. s.l. (including *P*. *strigosum* (Boenn.) Schur)	mak	28	B, D, O, M, P, SS, T, V, Z	293
Polygonaceae	*Rumex* sp.	štavljak	2	Z	275, 288
Ranunculaceae	*Ranunculus* cf *neapolitanus* Ten.	stopica, vučja stopica, medvjeđa šapa	3	Z	267, 289, 294
	*Ranunculus* sp.(?)	divlji selen	1	D	286
Valerianaceae	*Valerianella locusta* (L.) Laterrade*	matovilac	1	T	-
Unidentified taxa		berberuša	1	Z	249
		divlja salatina	1	V	251
		prči guzica	2	M	261
		salatuš	1	Z	274
		slično	1	D	317
		špinat divlji	1	S	319
		tušica	1	T	306
		divlji grašak	1	D	-

(?) uncertain identification based on small sterile specimens.

B - Biograd, C - Cavtat, D – Dubrovnik (2 markets), M - Makarska, O - Omiš, P - Ploče, T – Trogir, V - Vodice, S - Split, SS – Šibenik, Z – Zadar; * - a cultivated taxon; ** - commonly sold separately in most of the studied markets.

Two group taxa involving several botanical species should be pointed out. One is ž*utenica*/ž*ućenica* or *radič*/*radić*. This category encompasses a large number of Asteraceae (Cichorioideae) species. These are predominantly *Crepis* spp. (*C*. *biennis*, *C*. *zacintha*, *C*. *sancta*) but also other related genera (*Taraxacum officinale* Weber, *Leontodon taraxacoides* (Vill.) Mérat, *Reichardia picroides* (L.) Roth, *Cichorium intybus* L.). The respondents do not distinguish them well and usually cannot link the collected rosettes to the flowering forms. Some respondents even claimed that ž*utenica*/*radič* have no flowers and when these plants flower they stop being ž*utenica*/*radić*. Another collective name is *kozja brada* (literally *goat*’*s beard*) applied to at least three separate Asteraceae taxa (*Tragopogon* spp., *Scorzonera laciniata* L. and *Scorzonera villosa* Scop).

The sellers claim that they collected the plants in their home gardens or their vicinity, near the sea or in manure-fertilized arable fields. One of the sellers in Šibenik claimed that she waters the arable land on purpose to enhance the growth and germination of edible herbs.

The sellers state that the tradition of eating wild herbs has been alive as long as their grandparents remember. Until the 1960s herbs constituted a substantial part of people’s diet, but nowadays are used only occasionally, for example once a week as a side dish. They are boiled for 10–30 minutes, strained and seasoned heavily with olive oil and salt (e.g. 1 kg of wild herbs to 100–150 ml of olive oil). Sometimes also *pršut* (dried Croatian ham) is added. In the past (until the 1960s) the wild herbs were mixed with boiled potatoes, polenta or any other starchy products which were available. Another change our interviewers noticed is that now people have stopped collecting edible roots, they collect only leaves and stalks. For example before and during World War II, people were also eagerly seeking *Daucus carota* and *Eryngium* sp. (information from respondents in the market of Zadar and Vodice). In contrast to the thoroughly boiled wild vegetable mix, ž*ućenica*/*radič* Asteraceae are eaten raw or boiled for a very short time.

## Discussion

Why was such a rich heritage of wild vegetable gathering preserved in Dalmatia (and in Herzegovina) and not in most other Slavic countries [25,57] or for example in the neighbouring Hungary [58]? Even in northern Croatia the use of wild vegetables is much less widespread (authors’ personal observations). Two overlapping explanations are possible. One is that the high importance of wild vegetables is a remnant of the ancient Greek-Roman culture around the edge of the Mediterranean. Dalmatia was for a long time a part of the Roman Empire and the later Venetian state, and many towns were founded by the Greeks. *Radič* is actually a name of Greek origin (Cichorioideae plants are called similar names, e.g. ραδίκια in Greece). Another explanation is ecological-economical. Until the development of tourism, Dalmatia was the poorest part of former Yugoslavia – a rocky barren land where people had to eat wild produce to survive. The rocky dry hills were inhospitable to many cultivated vegetables, so people had to maintain knowledge of wild vegetables.

The results of the study show that collecting and selling wild green vegetables is still widespread in Dalmatia. At least 37 species are still gathered. Such a large number of wild greens is similar to wild vegetable mixes from Italy (e.g. [3,4,59]. However data supplied by Bakić and Popović [44], as well as the large number of species reported by Grlić [28] as sold in the markets of Dalmatia and our own participant observation and personal experiences (Table 2) suggest that a strong decrease in the number of the gathered taxa has appeared and the gathering is restricted to a fraction of the population. It should also be borne in mind that the list of taxa comes from a large area and on the scale of one market the number of taxa was much lower than in the previously mentioned Italian ethnobotanical studies.

**Table 2:** Comparison of available data on the use of wild green vegetables in Dalmatia and adjacent Bosnia-Herzegovina

	**Other data from Dalmatia and SW Herzegovina**	**Markets in Dalmatia in 2012**
*Allium ampeloprasum* L. (Liliaceae)	B, D, G, M, P, S	**
*Allium commutatum* Guss.	P	
*Allium subhirsutum* L.	P	
*Allium sphaerocephalon* L.	P, R	
*Allium roseum* L.	P	
*Allium schoenoprasum* L. (Liliaceae)	M	
*Allium vineale* L. (Liliaceae)	M	
*Amaranthus deflexus* L. (Amaranthaceae)	P	
*Amaranthus powellii* S.Watson (Amaranthaceae)	P	
*Amaranthus retroflexus* L. (Amaranthaceae)	G, P, R	
*Anchusa arvensis* (L.) M. Bieb. (Boraginaceae)	C	
*Anchusa* sp. (Boraginaceae)	C	
*Arum italicum* Mill. (Araceae)	B	
*Asparagus spp*. (mainly *Asparagus acutifolius* L.) (Asparagaceae)	B, G, C, P	
*Asparagus acutifolius* L. (Asparagaceae)	D	**
*Asparagus officinalis* L. (Asparagaceae)	M, R	
*Beta vulgaris* L. (Amaranthaceae) - wild	B, D, G, P, R	only cultivated
*Borago officinalis* L. (Boraginaceae)	G, M, S	*
*Brassica oleracea* L. (Brassicaceae)	D, G	only cultivated
*Bunias erucago* L. (Brassicaceae)	D, G, M	
*Capsella bursa*-*pastoris* L. (Brassicaceae)	G, M, R	
*Chenopodium album* L. (Chenopodiaceae)	M, P, R	
*Chenopodium urbicum* L. (Chenopodiaceae)	B	
*Chondrilla juncea* L. (Asteraceae)	M	
*Cichorium intybus* L. (Asteraceae)	B, D, G, M, S, P, R	*
*Cirsium arvense* L. (Asteraceae)	B, G	
*Clematis vitalba* L. (Ranunculaceae)	G, R	
*Crepis sancta* (L.) Babc. (Asteraceae)	C	*
*Crepis* spp. (Asteraceae)	C, D, M	**
*Crepis zacintha* (L.) Babc. (Asteraceae)	D	*
*Crithmum maritimum* L. (Apiaceae)	B, D, G, P, R	
*Daucus carota* L. (Apiaceae)	B, D, G, P, S, R	**
*Diplotaxis tenuifolia* (L.)DC. (Brassicaceae)	B, D, G, M, P	
*Erodium cicutarium* (L.) L'Hér. ex Aiton (Geraniaceae)	C, M	*
*Eruca sativa* Miller (Brassicaceae)	B, D, G	
*Eruca vesicaria* (L.) Cav.	P	
*Eryngium maritimum* L. and *E*. *campestre* L. (Apiaceae)	B, G, R	*
*Foeniculum vulgare* Mill. (Apiaceae)	B, C, D, G, M, P, S, R	**
*Geranium molle* L. (Geraniaceae)	C	
*Hirschfeldia incana* (L.) Lagr.-Foss. (Brassicaceae)	G	
*Hypochoeris radicata* L. (Asteraceae)	G	
*Lactuca perennis* L. (Asteraceae)	B, R	
*Lactuca serriola* L. (Asteraceae)	S	*
*Leontodon tuberosus* L. (Asteraceae)	B	
*Mentha aquatica* L. (Lamiaceae)	B	
*Ornithogalum umbellatum* L. (Liliaceae)	G, R	
*Papaver rhoeas* L. s.l. – including *Papaver strigosum* (Boenn.) Schur (Papaveraceae)	C, D, G, M, P, S	**
*Picris echioides* L. (Asteraceae)	D	**
*Pimpinella peregrina* L. (?) (Apiaceae)		*
*Plantago coronopus* L. (Plantaginaceae)	M, R	
*Portulaca oleracea* L. (Portulacaceae)	G, P, R	
*Ranunculus* cf *neapolitanus* Ten. (Ranunculaceae)		*
*Ranunculus muricatus* L. (Ranunculaceae)	C	
*Reichardia picroides* (L.) Roth. (Asteraceae)	G, M, P, S	*
*Rhagadiolus stellatus* (L.) Gaertn. (Asteraceae)	C, M	
*Rumex* spp. (Polygonaceae)	G, C, D, R	*
*Rumex patientia* L. (Polygonaceae)	M, R	
*Ruscus* spp. (Asparagaceae)	B, G, C, R	
*Salicornia herbacea* L. (Amaranthaceae)	G, R	
*Salvia verbenaca* L. (Lamiaceae)	C	
*Scorzonera laciniata* L. (Asteraceae)	P	*
*Scorzonera villosa* Scop. (?) (Asteraceae)	R	*
*Silene latifolia* Poir. (Caryophyllaceae)		**
*Silene vulgaris* (Mch.) Garcke ssp. *angustifolia* Hayek and related species (Caryophyllaceae)	B, G, M, P	
*Smilax aspera* L. (Smilacaceae)	G, R	
*Smyrnium olusatrum* L. (Apiaceae)		*
*Sonchus* spp. (Asteraceae), including *Sonchus asper* (L.) Hill ssp. *glaucescens* (Jord.) Ball, *S*. *oleraceus* L. ,S. *tenerrimus* L.	B, G, C, D, M, P, S, R	**
*Tamus communis* L. (Dioscoreaceae)	B, D, G, M, P, S, R	**
*Taraxacum megalorrhizon* (Forssk.) Hand.-Mazz. (Asteraceae)	B	
*Taraxacum officinale* Weber (Asteraceae)	B, D, G, M, P, R	**
*Taraxacum laevigatum* (Willd.) DC.	P	
*Tordylium apulum* L. (Apiaceae)	C	
*Tragopogon pratensis* L., *T. dubius* Scop. and *T*. *porrifolius* L. (Asteraceae)	B, D, G, M, P, S, R	*
*Urospermum picroides* F.W.Schmidt (Asteraceae)	C, D, G	**
*Urtica dioica* L. (Urticaceae)	D, S, R	
*Urtica pilulifera* L. (Urticaceae)	B, G	
*Urtica urens* L. (Urticacea)	P, R	
*Valerianella locusta* L. (Valerianaceae)	M	*
*Viola arvensis* Murr. (Violaceae)	M	

B – Bakić and Popović [44] (in this study it is unclear if the data is about eating green parts or underground organs, G – Grlić [28], C – Ćurčić [26], D - personal observations of Katija Dolina from Dubrovnik, one of the coauthors, M – personal observations of Tihomir Milicević from central Dalmatia and SW Herzegovina, one of the co-authors, P - personal observations of Marija Pandža from the island of Murter, R – Redžić [27] – beacuse this study encompasses a large number of species from the whole of Bosnia-Herzegovina, only the species listed by the other authors or found in the markets were confronted, S- Sardelić [45], ** – most commonly sold wild greens in Dalmatian markets in 2012 (this study), * – less commonly sold wild greens in Dalmatian markets in 2012 (this study).

An interesting issue is presented by differences in preparation techniques for wild greens encountered in the Mediterranean. In Italy wild greens are usually eaten fried (often with eggs), after the initial boiling, or made into a soup [3-6,59,60]. In southern France they are often eaten raw with dressing [61], whereas in Croatia most wild greens are boiled for a long time (usually nearly half an hour) and then dressed with olive oil (although some Asteraceae are eaten raw and *Asparagus* and *Tamus* are usually fried with eggs). Such way of preparation is similar to the Greek way of preparing *horta* (gr. χόρτα, *khorta*, *chorta*), traditional mixture of wild herbs still widely consumed in today's Greece [62]. This way of preparing wild greens (boiling for a long time and straining) may be the most primitive way of preparing wild greens, an adaptation for eating large amounts of them, since most toxins are removed. Eating wild greens as raw salads in France may be the result of a general trend of serving raw salads in France, and frying is possible only when larger amounts of oil are available.

The respondents mentioned a few times that they had noticed an increasing number of young health-oriented people (vegetarians etc.) buying the mix. This goes along with a similar trend in other European countries [63]. Thus an interesting phenomenon may be developing. The gradual decrease in the knowledge of plants is counteracted by specialized sellers who are the main holders of knowledge and suppliers of the plants to increasing circles of people. More local in-depth studies are needed to assess the relationships of the plants sold in markets with the plants known and used by the whole population of Dalmatia. It is however without doubt that the custom of selling wild vegetable mixes has a long uninterrupted tradition and represents a part of the traditional Dalmatian heritage, although throughout the last several decades it has changed from a dish enriched with starchy foods to a separate salad or side-dish.

At the moment very few restaurants on the coast sell the wild vegetable mixes (e.g. a hotel in Makarska), however increasing numbers of people in Zagreb, the capital of Croatia, buy the mixes imported from Dalmatia and sold in Zagreb markets.

It is surprising that some wild vegetables, abundant in the study area and used in other parts of the Mediterranean, and even among Croatian population in the neighboring Bosnia and Herzegovina, e.g. *Malva* spp. and *Silene vulgaris* (Moench) Garcke were not recognized as edible plants by the sellers (they were presented to them in a fresh state, gathered from the neighbourhood of the markets). Similarly *Stellaria media* (L.) Vill. is not eaten. It is often present as an unintentional admixture in the mix and every time respondents said that this is only *trava* (herb/grass) and threw it out of the mix, ashamed of the “contamination”.

Polish economic botanists Rostafiński [64] and Maurizio [65] were interested in the process by which dishes from wild vegetables gradually turned into dishes made of domesticated greens. The former found that the northern Slavic soup *borsch* (Polish *barszcz*) was originally made with *Heracleum sphondylium* L. but gradually throughout the 17-19^th^ century was turned into a soup dominated by the cultivated beet *Beta vulgaris L*. subsp. *vulgaris* L. Łuczaj [25] in his review of changes in the use of wild vegetables in Poland found more such functional pairs of wild greens and the cultivated greens, which replaced them: ground ivy *Glechoma hederacea* L., versus parsley *Petroselinum crispum* (Mill.) Fuss as well as fat-hen *Chenopodium album* L., versus spinach *Spinacia oleracea* L. and *Brassica* spp. It is not unlikely that a similar process occurs in Croatia, as several cultivated vegetables are added into *mišanca* now. It should be noted that the majority of beet sold in the market is now the cultivated form, whereas in the past more wild beet was sold.

Although most wild vegetables sold in the markets of Dalmatia are the species commonly consumed in southern Europe, the use of a few of the species, namely *Scorzonera laciniata*, *Urospermum picroides* and *Ranunuclus neapolitanus* is not reported by any major directories of edible plants (e.g. [66-70]).

## Conclusions

Wild edible plant mixes are sold widely in Dalmatia and present in every market. However they are relatively species-poor, usually composed of just a few species of wild and, to a lesser extent, cultivated vegetables. The selling of these mixes has a long, continuous tradition remembered by respondents since childhood. Signs of both the degeneration and revival of the tradition are present, however a decrease in the general knowledge of plants among the population of Dalmatia is obvious. Further studies are needed to establish the relationship of the market-sold mixes to the choice of plants gathered for individual use, although it is probably similar.

## Competing interests

The authors declare that they have no competing interest.

## Authors’ contributions

MZK and ŁŁ initiated the study and gathered the literature. ŁŁ performed the interviews and identified most plants. MZK, TM, KD and MP enriched the study with personal long term observations on the subject and helped to draft the results and discussion sections, as well as to identify some taxa. All the authors read and discussed the final form of the article.

## References

[B1] Rivera D Obón C Inocencio C Heinrich M Verde A Fajardo J Llorach R The ethnobotanical study of local Mediterranean food plants as medicinal resources in Southern Spain J Physiol Pharmacol 2005 56 Suppl 97 114 15800388

[B2] Hadjichambis A Paraskeva-Hadjichambi D Della A Giusti ME De Pasquale C Lenzarini C Censorii E Gonzales-Tejero MR Sanchez-Rojas CP Ramiro-Gutierrez JM Wild and semi-domesticated food plant consumption in seven circum-Mediterranean areas Int J Food Sci Nutr 2008 59 5 383 414 10.1080/09637480701566495 18979618

[B3] Paoletti MG Dreon AL Lorenzoni GG Pistic, traditional food from Western Friuli, N.E. Italy Econ Bot 1995 49 1 26 30 10.1007/BF02862273

[B4] Pieroni A Gathered wild food plants in the upper valley of the Serchio River (Garfagnana), Central Italy Econ Bot 1999 53 3 327 341 10.1007/BF02866645

[B5] Pieroni A Nebel S Quave C Munz H Heinrich M Ethnopharmacology of liakra: traditional weedy vegetables of the Arbereshe of the Vulture area in southern Italy J Ethnopharmacol 2002 81 2 165 185 10.1016/S0378-8741(02)00052-1 12065148

[B6] Pieroni A Quave C Nebel S Heinrich M Ethnopharmacy of the ethnic Albanians (Arbereshe) of northern Basilicata, Italy Fitoterapia 2002 73 3 217 241 10.1016/S0367-326X(02)00063-1 12048017

[B7] Guarrera PM Food medicine and minor nourishment in the folk traditions of Central Italy (Marche, Abruzzo and Latium) Fitoterapia 2003 74 515 544 10.1016/S0367-326X(03)00122-9 12946715

[B8] Guarrera PM Salerno G Caneva G Food, flavouring and feed plant traditions in the Tyrrhenian sector of Basilicata, Italy J Ethnobiol Ethnomed 2006 2 37 10.1186/1746-4269-2-37 16959031 PMC1592457

[B9] Nebel S Heinrich M Ta chòrta: a comparative ethnobotanical-linguistic study of wild food plants in a graecanic area in Calabria, Southern Italy Econ Bot 2009 63 1 78 92 10.1007/s12231-008-9069-9

[B10] Nebel S Pieroni A Heinrich M Ta chorta: wild edible greens used in the Graecanic area in Calabria, Southern Italy Appetite 2006 47 3 333 342 10.1016/j.appet.2006.05.010 16843569

[B11] Ghirardini MP Carli M Del Vecchio N Rovati A Cova O Valigi F Agnetti G Macconi M Adamo D Traina M Laudini F Marcheselli I Caruso N Gedda T Donati F Marzadro A Russi P Spaggiari C Bianco M Binda R Barattieri E Tognacci A Girardo M Vaschetti L Caprino P Sesti E Andreozzi G Coletto E Belzer G Pieroni A The importance of a taste: a comparative study on wild food plants consumption in twenty-one local communities in Italy J Ethnobiol Ethnomed 2007 3 22 10.1186/1746-4269-3-22 17480214 PMC1877798

[B12] Bonet MA Vallès J Use of non-crop food vascular plants in Montseny biosphere reserve (Catalonia, Iberian Peninsula) Int J Food Sci Nutr 2002 53 225 248 10.1080/09637480220132841 11951586

[B13] Tardío J Pardo de Santayana M Morales R Ethnobotanical review of wild edible plants in Spain Bot J Linnean Soc 2006 152 27 72 10.1111/j.1095-8339.2006.00549.x

[B14] Tardío J Pascual H Morales R Wild food plants traditionally used in the province of Madrid Econ Bot 2005 9 2 122 136

[B15] Pardo-de-Santayana M Tardío J Morales R The gathering and consumption of wild edible plants in the Campoo (Cantabria, Spain) Int J Food Sci Nutr 2005 56 7 529 542 10.1080/09637480500490731 16503563

[B16] Pardo-de-Santayana M Tardío J Blanco E Carvalho AM Lastra JJ Miguel ES Morales R Traditional knowledge of wild edible plants used in the northwest of the Iberian Peninsula (Spain and Portugal): a comparative study J Ethnobiol Ethnomed 2007 3 27 10.1186/1746-4269-3-27 17555572 PMC1904191

[B17] Tardío J Pardo-de-Santayana M, Pieroni A, Puri R Spring is coming: the gathering and consumption of wild vegetables in Spain Ethnobotany in the New Europe: people, health and wild plant resources 2010 Oxford-New York: Berghahn Books 211 238

[B18] Menendez-Baseta G Aceituno-Mata L Tardío J Reyes-García V Pardo-de-Santayana M Wild edible plants traditionally gathered in Gorbeialdea (Biscay, Basque Country) Genet Resour Crop Evin press

[B19] Leonti M Nebel S Rivera D Heinrich M Wild gathered food plants in the European Mediterranean: a comparison analysis Econ Bot 2006 60 130 142 10.1663/0013-0001(2006)60[130:WGFPIT]2.0.CO;2

[B20] Della A Paraskeva-Hadjichambi D Hadjichambis AC An ethnobotanical survey of wild edible plants of Paphos and Larnaca countryside of Cyprus J Ethnobiol Ethnomed 2006 2 34 10.1186/1746-4269-2-34 16995927 PMC1599709

[B21] Forbes MHC Dimen M, Friedl E Gathering in the Argolid: a Subsistence Subsystem in a Greek Agricultural Community Regional Variation in Modern Greece and Cyprus: Toward a Perspective on the Ethnography of Greece 1976 New York: Annals of the New York Academy of Sciences 251 264 268

[B22] Dogan Y Traditionally used wild edible greens in the Aegean Region of Turkey Acta Soc Bot Pol 2012 81 4 329 342

[B23] Ali-Shtayeh MS Jamous RM Al-Shafie' JH Elgharabah WA Kherfan FA Qarariah KH Khdair IS Soos IM Musleh AA Isa BA Herzallah HM Khlaif RB Aiash SM Swaiti GM Abuzahra MA Haj-Ali MM Saifi NA Azem HK Nasrallah HA Traditional knowledge of wild edible plants used in Palestine (Northern West Bank): a comparative study J Ethnobiol Ethnomed 2008 4 13 10.1186/1746-4269-4-13 18474107 PMC2396604

[B24] Łuczaj Ł Archival data on wild food plants used in Poland in 1948 J Ethnobiol Ethnomed 2008 4 4 10.1186/1746-4269-4-4 18218132 PMC2275233

[B25] Łuczaj Ł Changes in the utilization of wild green vegetables in Poland since the 19th century: a comparison of four ethnobotanical surveys J Ethnopharmacol 2010 128 395 404 10.1016/j.jep.2010.01.038 20097282

[B26] Ćurčić V Narodno ribarstvo u Bosni i Hercegovini. Part 2 Glasnik Zemaljskog Muzeja u Bosni i Hercegovini 1913 25 464 465

[B27] Redžić S Wild edible plants and their traditional use in the human nutrition in Bosnia‐Herzegovina Ecol Food Nutr 2006 45 3 189 232 10.1080/03670240600648963

[B28] Grlić L Enciklopedija samoniklog jestivog bilja 2005 Rijeka: Ex Libris

[B29] Moszyński K O sposobach badania kultury materialnej Prasłowian 1962 Wrocław: Zakład Narodowy im. Ossolińskich

[B30] Redžić S Barudanović S Pilipović S Wild mushrooms and lichens used as human food for survival in war conditions; Podrinje - Zepa region (Bosnia and Herzegovina, W. Balkan) Hum Ecol Rev 2010 17 2 175 181

[B31] Redžić S Use of wild and semi-wild edible plants in nutrition and survival of people in 1430 days of siege of Sarajevo during the war in Bosnia and Herzegovina (1992–1995) Coll Antropol 2010 34 2 551 570 20698130

[B32] Pieroni A Giusti ME Münz H Lenzarini C Turković G Turković A Ethnobotanical knowledge of the Istro-Romanians of Žejane in Croatia Fitoterapia 2003 74 7–8 710 719 14630181 10.1016/j.fitote.2003.06.002

[B33] Pieroni A Local plant resources in the ethnobotany of Theth, a village in the Northern Albanian Alps Genet Resour Crop Ev 2008 55 1197 1214 10.1007/s10722-008-9320-3

[B34] Jarić S Popović Z Mačukanović-Jocić M Djurdjević L Mijatović M Karadžić B Mitrović M Pavlović P An ethnobotanical study on the usage of wild medicinal herbs from Kopaonik Mountain (Central Serbia) J Ethnopharmacol 2007 111 1 160 175 10.1016/j.jep.2006.11.007 17145148

[B35] Pieroni A Quave C Giusti ME Cross-cultural ethnobiology in the Western Balkans: medical ethnobotany and ethnozoology among Albanians and Serbs in the Pešter plateau, Sandžak, southwestern Serbia Hum Ecol 2011 39 333 349 10.1007/s10745-011-9401-3

[B36] Mustafa B Hajdari A Krasniqi F Hoxha E Ademi H Quave CL Pieroni A Medical ethnobotany of the Albanian Alps in Kosovo J Ethnobiol Ethnomed 2012 8 6 10.1186/1746-4269-8-6 22284581 PMC3285519

[B37] Mustafa B Hajdari A Pajazita Q Syla B Quave CL Pieroni A An ethnobotany survey of the Gollak region, Kosovo Genet Resour Crop Ev 2012 59 739 754 10.1007/s10722-011-9715-4

[B38] Šarić-Kundalić B Dobeš C Klatte-Asselmeyer V Saukel J Ethnobotanical survey of traditionally used plants in human therapy of east, north and north-east Bosnia and Herzegovina J Ethnopharmacol 2011 133 3 1051 1076 10.1016/j.jep.2010.11.033 21094241

[B39] Lovrić AZ Rac M Mileković MH Diversity of old-Croatian names for seaweeds and maritime nature in the Adriatic Islands Natura Croatica 2002 11 4 455 477

[B40] Nikolić T Rešetnik I Plant uses in Croatia Phytologia Balcanica 2007 13 2 229 238

[B41] Pieroni A Giusti ME The remedies of the folk medicine of the Croatians living in Čičarija, Northern Istria Collegium Antropol 2008 32 623 627 18756920

[B42] di Tizio A Łuczaj ŁJ Quave CL Redžić S Pieroni A Traditional food and herbal uses of wild plants in the ancient South-Slavic diaspora of Mundimitar/Montemitro (Southern Italy) J Ethnobiol Ethnomed 2012 8 21 10.1186/1746-4269-8-21 22672636 PMC3484038

[B43] Grlić L Enciklopedija samoniklog jestivog bilja 1986 Zagreb: August Cesarec

[B44] Bakić J Popović M Nekonvencionalni izvori u ishrani na otocima i priobalju u toku NOR-a 1983 Beograd: Izd. Mornaričkog glasnika 49 55

[B45] Sardelić S Samoniklo jestivo bilje – mišanca, gruda, parapač… wild edible herbs – Mišanca, Gruda, Parapač Etnološka istraživanja 2008 1 12/13 387 396 http://hrcak.srce.hr/37036

[B46] Nguyen MLT Doherty KT Wieting J Market survey research: a model for ethnobotanical education Ethnobot Res Appl 2008 6 87 92

[B47] Bye RA Linares E The role of plants found in the Mexican markets and their importance in ethnobotanical studies J Ethnobiol 1983 3 1 13

[B48] Pemberton RW Lee NS Wild food plants in South Korea; market presence, new crops, and exports to the United States Econ Bot 1996 50 57 70 10.1007/BF02862113

[B49] Xu Y-K Tao G-D Liu H-M Yan K-L Dao X-S Wild vegetable resources and market survey in Xishuangbanna, Southwest China Econ Bot 2004 58 4 647 667 10.1663/0013-0001(2004)058[0647:WVRAMS]2.0.CO;2

[B50] Szulczewski JW Łysiak W Pieśń bez końca: Zbiór tekstów folkorystyczno-etnograficznych 1996 PSO, Poznań

[B51] Łuczaj ŁJ Plant identification credibility in ethnobotany: a closer look at Polish ethnographic studies J Ethnobiol Ethnomed 2010 6 36 10.1186/1746-4269-6-36 21167056 PMC3022638

[B52] Šugar I Hrvatski biljni imenoslov. Nomenclator botanicus Croaticus 2008 Zagreb: Matica Hrvatska

[B53] Łuczaj Ł Nieroda Z Collecting and learning to identify edible fungi in Southeastern Poland: age and gender differences Ecol Food Nutr 2011 50 319 336 10.1080/03670244.2011.586314 21888599

[B54] Łuczaj ŁJ Kujawska M Botanists and their childhood memories: an under-utilized expert source in ethnobotanical research Bot J Linn Soc 2012 168 334 343 10.1111/j.1095-8339.2011.01205.x

[B55] Tutin TG Heywood VH Burges DM Moore DH Valentine SM Walters SM Webb DA Flora Europaea, Vol. 1–5 Cambridge and London: The University Press 1964–1980

[B56] The International Plant Name Index http://ipni.org/ipni/plantnamesearchpage.do Accessed 30 October 2012

[B57] Łuczaj Ł Ethnobotanical review of wild edible plants of Slovakia Acta Soc Bot Pol 2012 81 4 245 255

[B58] Dénes A Papp N Babai D Czúcz B Molnár Z Wild plants used for food by Hungarian ethnic groups living in the Carpathian Basin Acta Soc Bot Pol 2012 81 4 381 396

[B59] Turner NJ Łuczaj ŁJ Migliorini P Pieroni A Dreon AL Sacchetti L Paoletti MG Edible and tended wild plants, traditional ecological knowledge and agroecology Cr Rev Plant Sci 2011 30 198 225 10.1080/07352689.2011.554492

[B60] Pieroni A Nebel S Santoro RF Heinrich M Food for two seasons: culinary uses of non-cultivated local vegetables and mushrooms in a south Italian village Int J Food Sci Nutr 2005 56 245 272 10.1080/09637480500146564 16096136

[B61] Marco C Chauvet M Mathez J Ubaud J Passama L Garrone B Molina J Cornillon M Martin P Wotan JM Les salades sauvages. L'Ensalada champanèla 2003 Les Ecologistes de L'Euzière, Sant Jean de Cuculles

[B62] Anonymous Greek Greens (Horta) Authentic Greek Recipeshttp://realgreekrecipes.blogspot.gr/2010/03/greek-greens-horta.html Accessed 20 June 2012

[B63] Łuczaj Ł Pieroni A Tardío J Pardo-de-Santayana M Sõukand R Svanberg I Kalle R Wild food plant use in 21st century Europe: the disappearance of old traditions and the search for new cuisines involving wild edibles Acta Soc Bot Pol 2012 81 4 359 370

[B64] Rostafiński J O nazwach oraz użytkach ćwikły, buraków i barszczu 1916 Kraków: Akademia Umiejętności

[B65] Maurizio A Geschichte unserer Pflanzennahrung, von den Urzeiten bis zur Gegenwart 1927 Berlin: Paul Parey

[B66] Hedrick UP Sturtevant’s Edible Plants of the World 1972 New York: Dover Publications 1919

[B67] Tanaka T Cyclopaedia of Edible Plants of the World 1976 Tokyo: Keigaku Publishing

[B68] Kunkel G Plants for human consumption: an annotated checklist of the edible phanerogams and ferns 1984 Koenigstein: Koelz Scientific Books

[B69] Vaughan JG Geissler CA The new Oxford book of food plants 2009 Oxford: Oxford University Press

[B70] Plants for a Future http://pfaf.org

